# Student support systems for undergraduate medical students during the COVID-19 pandemic: a systematic narrative review of the literature

**DOI:** 10.1186/s12909-021-02791-9

**Published:** 2021-06-22

**Authors:** Ali Ardekani, Seyed Ali Hosseini, Parinaz Tabari, Zahra Rahimian, Afrooz Feili, Mitra Amini, Arash Mani

**Affiliations:** 1 Student Research Committee, Shiraz University of Medical Sciences, Shiraz, Iran; 2 Clinical Education Research Center, Shiraz University of Medical Sciences, Shiraz, Iran; 3 Research Center for Psychiatry & Behavioral Sciences, Shiraz University of Medical Sciences, Shiraz, Iran

**Keywords:** Medical education, Mentors, Mentoring, COVID-19, Medical students

## Abstract

**Background:**

The coronavirus disease 2019 (COVID-19) pandemic has boosted medical students’ vulnerability to various problems. Given the stressful nature of medical disciplines, considerable attention must be paid to student support systems during pandemics. This study aimed to review the current literature regarding medical student support systems systematically.

**Methods:**

We performed a systematic review of six databases and grey literature sources in addition to a hand search in the references of the articles on April 5, 2021. We included all studies about support for undergraduate medical students delivered in response to the COVID-19 pandemic. In conducting this review, we used the Preferred Reporting Items for Systematic Reviews and Meta-Analyses (PRISMA) statement.

**Results:**

A total of 3646 articles were retrieved from the databases, and 16 additional papers were extracted from other sources. After removing duplicates, we screened 2434 titles and abstracts according to our criteria. Among them, 32 full-text articles were assessed for eligibility. Ultimately, 10 studies were included for review. We identified two major themes: (a) academic support and (b) mental health support. All of the included studies utilized online methods whether for transitioning from previous support systems or developing novel approaches. Students and faculty members seemed to be receptive to these new systems. Despite indicating outstanding program outcomes, most studies merely described the positive effects of the program rather than providing a precise evaluation.

**Conclusion:**

There are several methods of supporting medical students who are experiencing unprecedented changes in their educational trajectory. Due to substantial differences in undergraduate medical education in different regions of the world, cultural and contextual-oriented support is indispensable for developing a safe learning environment. Future research should investigate the question of the extent to which online support can supersede in-person strategies.

**Supplementary Information:**

The online version contains supplementary material available at 10.1186/s12909-021-02791-9.

## Background

Following the outbreak of the severe acute respiratory syndrome coronavirus 2 (SARS-CoV-2) in China, and the rapid spread of its consequent disease called COVID-19 worldwide, diverse social aspects of human life have been affected [1, 2]. As one of the most critical facets of social life, education has encountered many challenges considering its interactive nature [3]. For instance, medical education has faced plenty of issues due to the need for students to communicate with patients and, on the other hand, the responsibility of faculty staff to ensure the health of students, which is of paramount concern [4]. Students’ daily commute to teaching hospitals leads to the spread of the virus. Accordingly, the cancellation of instructional programs and the use of virtual teaching methods have been put on the agenda [5]. Besides physical health concerns, the stressful nature of the medical disciplines exerts a great deal of psychological and social stress on students during their study period [6]. Research has discovered that medical students are more likely than the rest of the population to suffer from mental illnesses, with a greater risk of depression and suicidal ideas [7]. Notably, medical trainees fighting the pandemic on the frontlines are under much psychological stress [8]. Aside from the previously mentioned issues, COVID-19-related challenges to formal methods of clinical teaching have raised the signs of adverse mental well-being among medical students globally [9]. All of the mentioned problems highlight the importance of providing medical students with academic and personal support, thereby facilitating their success during their training [10, 11]. The ultimate goal of student support systems is not merely to empower a student in academic and clinical competencies but also to develop all aspects of the student’s character as a whole [12]. One way for students to adapt to new situations is to use a type of student support system called mentoring [13]. In a systematic review in 2018, Akinla et al. concluded that near-peer mentoring programs for first-year medical students are among the most effective methods of supporting them for professional and academic achievement [13].

Of course, it should be noted that supporting students is not limited to mentoring and includes a wide range of methods of support for student education, mental health, and even decision-making [12]. An integrated model consisting of dedicated counseling sessions for medical students, wellness group activities, online courses, and collaboration with student health services for referral (e.g., psychiatric counseling) is employed at Florida International University to reduce burnout and depression as byproducts of studying medicine [14]. One study calls for developing stress management programs through training workshops to prevent mental health conditions in medical schools given the high prevalence of such disturbances among medical students [15]. Considering the extra psychological burden that the current pandemic has put on medical students, student support systems’ employment seems more essential than ever before [16–19]. Lessons learned from previous pandemics regarding these systems can be considered when devising the related programs [20]. Recently, increased attention has been directed toward supporting medical students because, in these unprecedented times, they are more vulnerable than ever before to a wide range of adversities [21].

The publication of articles after the outbreak of COVID-19 is growing exponentially [22]. A number of studies have introduced a diversity of new methods to support and educate students. However, these methods need to be comprehensively reviewed before being utilized by medical schools in the unanticipated future. In the present study, we intended to perform a comprehensive systematic review of the ways of supporting undergraduate medical students during the COVID-19 pandemic. It is hoped that by completing this review, the path of supporting students will be further paved.

## Methods

In conducting this review, the Preferred Reporting Items for Systematic Reviews and Meta-Analyses (PRISMA) protocol was followed [23].

### Search strategy

In June 2020, the initial search was done to investigate suitable keywords and reach relevant results. The first round of search and screening was conducted at the end of July 2020. Given the rapid expansion of literature in the COVID-19 era, we decided to re-conduct the search in order to access the latest evidence available on the topic. The final search was done on April 5, 2021, using a more specific search strategy representing the “COVID-19 pandemic”, “medical education,” and “student support”. Some other entry terms and synonyms were utilized, along with the mentioned keywords. The PubMed, Embase, Scopus, Web of Science, ERIC, and Cochrane Library databases were explored using word clusters and proper Boolean operators (AND; OR). Each database was investigated with an appropriate search strategy (Supplementary file 1). To access the grey literature, the first 100 results of Google Scholar were also included. Additionally, a hand search in the retrieved articles’ reference lists was performed. All citations were imported into EndNote X9 software (Clarivate Analytics, USA), and duplicates were removed. Then, we entered the citations into the Rayyan (available at https://www.rayyan.ai), which is a free web app that facilitates the screening process for systematic reviews [24].

### Study selection

This study aimed to review any kind of student support employed by medical universities in response to the unprecedented changes that arose from the pandemic. Titles and abstracts of all articles found were screened by A.A. and Z.R. independently. In the absence of an abstract, the full text of the study was used. The inclusion criteria were: studies that mentioned student support system programs in any stage of undergraduate medical education; articles published after December 1st 2019; and studies describing student support systems explicitly used in response to the COVID-19 pandemic. The exclusion criteria were: opinion pieces and other studies involving no actual changes; studies that described support systems that were not used in response to the COVID-19 pandemic; and articles that did not discuss support systems in undergraduate medical education. No exclusion occurred due to article language or country of publication. After initial screening, relevant articles were reached in full text and investigated by A.A. and P.T. independently for eligibility. In the process of screening, all disagreements between authors were solved by discussion or group consensus with a third party (M.A. or A.M.).

### Data extraction

All eligible studies were reviewed by A.F. and S.A.H. independently. Where there was a discrepancy between reviewers, the study was reviewed by the most expert author (M.A. or A.M.), and a consensus was reached through discussion. A Microsoft Excel (Microsoft, Redmond, WA, U.S.) spreadsheet was used for data collection. The data extracted included the author(s), the country of the project, the support system’s goal, the intervention, and the outcomes.

### Critical appraisal

A checklist containing 11 items introduced by Buckley et al. [25] in 2009 was utilized to appraise the studies (Supplementary 1). If a study met seven out of 11 criteria, it was considered as a high-quality study. Although all studies were appraised precisely, none were excluded due to having low quality. P.T. and S.A.H. appraised the studies independently; whenever there was a dispute, M.A. or A.M. was consulted, and a consensus was reached.

## Results

### Search results

**Fig. 1:**
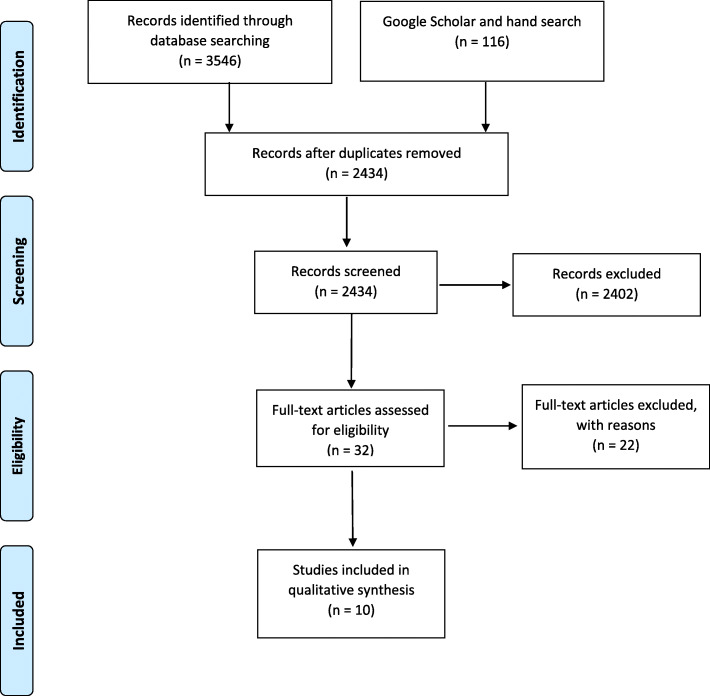
PRISMA flow-diagram for systematic reviews

On April 5, 2021, the final database search yielded 3662 articles, consisting of 441 citations in PubMed, 513 in Embase, 459 in Scopus, 183 in WOS, 9 in the Cochrane Library, 1941 in ERIC, and 100 in Google Scholar. Also, 16 additional studies were included by a hand search in the references and relevant journals in the field of medical education (Fig. 1). After removing duplicates, 2434 titles and abstracts screened for the first time resulted in 32 relevant studies. The full texts of 32 articles were retrieved and examined. Finally, 10 articles were found to be eligible. In terms of geographical distribution, three were from Germany, two from the United Kingdom (UK), two from Singapore, one from the United States, one from Brazil, and one from Iran. A summary of the findings is presented in Table 1.

**Table 1:** Summary of the reviewed studies

Author(s)	Country	Goal	Intervention (n = participants)	Outcomes
Ashokka et al. [26]	Singapore	To provide students with social, educational, emotional, and material support.	Support was provided to quarantined students through volunteer groups, peer support systems, and faculty staff, some of whom visited the student dormitories while maintaining personal protection. Both educational and emotional support was provided, with relief packages also being dispensed among the students. Online communication platforms formed the basis of the majority of the social support and teaching services.	By obtaining informal feedback from the parties involved, the researchers found that a smooth transition to online communication was made, though technical difficulties (e.g., poor internet bandwidth) and concerns regarding the need for real-life student-patient interactions were put forth by both students and staff.
Blasco et al. [27]	Brazil	Maintaining mental and emotional stability	Short videos were provided in order to address emotional and mental issues and coping strategies. The videos were adapted from cinema movies.	Early feedback obtained via social media were analyzed by a qualitative approach, indicating the positive impact of the program.
Gernert et al. [28]	Germany	Adapting to the unexpected halt in traditional mentoring and fixing the resulting confusion of many students.	Adapting the existing mentoring program to digital platforms through three different events: 1. How To Klink (*n* = 120); 2. FachartzDuell (*n* = 105); and 3. Eight sessions of *Auf ein gesprach mit* (AEGM), which provided support in clinical studies and career counseling.	A survey showed that the participating students had a high degree of satisfaction. Also, online support platforms were welcomed as well as in-person strategies.
Guse et al. [29]	Germany	To investigate the effect of mentoring on psychological issues and perspectives on the educational status of students.	General mentoring program (g-mentoring) for all volunteer students and e-mentoring for students with outstanding course outcomes and scientific interest (from year two onwards). Outcomes were evaluated with the Patient Health Questionnaire (PHQ-4) and by determining the perspectives on educational status using self-created objectives in comparison with students who did not participate in mentoring programs (*n* = 543).	Trainees in e- and g- mentoring groups had lower levels of mental issues. Most of the participants (55%) were concerned about their educational status in the pandemic era. More students in the e-mentoring group reported being “as worried or unworried as before” about the educational status. The majority of students, regardless of mentoring, reported a drop in motives for education.
Hodgson & Hagan [30].	United Kingdom	To offer online virtual support to students.	Student support groups were transitioned to a virtual support system using the Microsoft Teams software (Microsoft, Redmond, WA, U.S.) at two UK universities during the COVID-19 pandemic.	Positive feedback was obtained regarding the new support system.
Huddart et al. [31]	United Kingdom	To resolve medical students’ ambiguity about incoherent information and exhibit student initiatives.	A one-hour national Twitter-based discussion on the uncertainties, concerns, and initiatives of UK medical students concerning the COVID-19 was done.	Some of the students’ critical concerns included the fear of burnout, the necessity of adequate supervision, and the need for personal protective equipment. Furthermore, students shared uncertainty regarding whether they could obtain the required clinical skills through online learning systems.
Lee et al. [32]	Singapore	To provide academic support to medical students, thereby helping them overcome problems related to new educational programs during the COVID-19 pandemic.	A coaching program was devised using the master adaptive learning (MAL) framework to build on medical students’ abilities and provide them with much-needed support during the COVID-19 pandemic. The framework consisted of the four stages of planning, learning, assessing, and adjusting.	The program led to significant improvements in the students’ academic performance besides providing them with an excellent support network during the troubling conditions.
Rastegar Kazerooni et al. [33]	Iran	To improve students’ coping skills and mental preparedness in the face of the pandemic.	Junior medical students underwent near-peer mentoring by senior students and expert faculty members using a social media platform. (*n* = 371)	The survey questionnaire indicated a positive impact on the professional growth of the juniors. The program helped them to adjust to the unprecedented conditions. Nonetheless, the desire for non-virtual face to face consultations prevailed
Stetson et al. [34]	USA	To promote professional identity formation and reduce anxiety, fear, and stress during the COVID-19 pandemic.	The Zoom application (Zoom Video Communications Inc., USA) was used to facilitate small group-based guided reflection to promote professional identity formation by mitigating anxiety, fear, and stress among medical students of the San Francisco School of Medicine.	The students felt that by allowing them to discuss their thoughts and emotions with their peers, the program reduced their isolation and normalized their reactions, ultimately reducing stress and anxiety.
Zibold et al. [35]	Germany	To adapt to online methods for mentoring medical students.	Monthly training for peer mentors via Zoom, weekly counseling sessions with the mentoring program coordinator for students, and developing a new activity (“PubQuiz”) to reduce the psychological burden and increase collaborations between mentees and mentors (*n* = 35).	Positive feedback was provided from the program users. Also, users asked for the continuation of the online program as well as in-person ones.

### Critical appraisal

We appraised all studies against the Buckley et al. [25] checklist; however, none of the included studies met at least seven out of the 11 items of the checklist to be considered as high-quality studies. The low quality of studies has been mentioned in a previous review in the context of medical education [36]. Most studies tend to merely describe the educational intervention rather than comprehensively evaluating its outcomes.

### Summary of the included articles

Although it is challenging to separate the described support strategies, the eligible studies can be categorized into two major themes:

- Academic support to help medical students cope with new educational changes during the COVID-19 pandemic.
- Mental health support for students during the COVID-19 pandemic.

### Academic support

Three papers reported new adaptations to provide academic support to medical students. These articles attempted to identify current issues and ease the transition to new circumstances. In the study conducted by Huddart et al. [31], the outcomes of a one-hour national Twitter-based discussion on the uncertainties, concerns, and initiatives of UK medical students in relation to COVID-19 were presented. Some of the students’ critical concerns included the fear of burnout, the necessity of adequate supervision, and the need for personal protective equipment. Furthermore, students shared uncertainty regarding whether they could obtain the required clinical skills through online learning systems. The initiatives discussed included clinical volunteering and non-clinical volunteer work like awareness campaigns and community members’ assistance. In a Singapore-based study directed by Lee et al. [32], a coaching program was devised using the master adaptive learning framework to build on medical students’ abilities and provide them with much-needed support during the COVID-19 pandemic. The program involved both academic coaches, who tracked and analyzed the medical students’ performance while also examining their methods of studying and learning through regular 30-min discussions, and faculty staff, who addressed the students’ specific content-based needs. The students found the program’s proactive support highly helpful, with many acknowledging that the individualized goal-based studying strategies and short-interval follow-up sessions boosted their motivation, accountability, reflectiveness, and studying efficiency. Furthermore, coaches provided assistance when the students lost their motivation over time and found it challenging to adhere to their plans for studying; a holistic approach was employed that took into account the problems of the study plan as well as the mental well-being and self-care of the student. In another study, the coordinated national responses of medical teaching institutions during the COVID-19 pandemic were discussed by Ashokka et al. [26], with a particular emphasis being placed on the need to sustain medical education. One central theme discussed was the enhancement of university support systems. Online communication platforms formed the basis of the majority of the social support and teaching services. Furthermore, each sub-cohort or clinical group of students had a leader for representation and coordination in the student network system. In this network, the plans for digital changes in the teaching environment, the students’ expectations, the administrative requirements, the online code of conduct, and the support systems available were presented. Furthermore, students could send their inquiries, concerns, and ideas to the COVID-19 response team through a feedback system.

### Mental health support

Four studies concerned psychological issues. In the study of Rastegar Kazerooni et al. [33], a social media platform was established in Iran for near-peer mentoring during the COVID-19 pandemic. Ten senior medical students who had been thoroughly trained in peer mentoring were supervised by the medical education faculty staff in their efforts to mentor 371 juniors through the online platform. During discussions about the junior students’ concerns and needs, several key recommendations were passed on by the senior peers, including stress management and relaxation, exercise, virtual contact with peers and family, and time management during the quarantine period. According to a survey of the participants, the program positively impacted the professional growth of the juniors and helped them to adjust to the unprecedented conditions. Nonetheless, the desire for non-virtual face to face consultations prevailed. In a related article, Stetson et al. [34] discussed their experience with the use of the Zoom application (Zoom Video Communications Inc., USA) to provide small, group-based, guided reflection sessions aimed at promoting professional identity formation by mitigating anxiety, fear, and stress among medical students of the San Francisco School of Medicine during the COVID-19 pandemic. The students felt that by empowering them to discuss their thoughts and emotions with their peers, the program reduced their isolation and normalized their reactions, ultimately reducing both stress and anxiety. In another study, Hodgson and Hagan [30] described their experience with the transition of student support groups to a virtual support system using the Microsoft Teams software (Microsoft, Redmond, WA, U.S.) at two UK universities during the COVID-19 pandemic. Institutional subscriptions were obtained for the software, with free access to all students and staff via smartphones and computers. The software facilitated both one-to-one and group communication via video, audio, and text. A key finding was that the quality of the experience for both students and staff was enhanced when video calls were made instead of audio calls as the parties involved could have a better conversation when they could see one another. Furthermore, scheduled video calls were useful in maintaining an organized structure, which was essential for keeping a routine, maintaining well-being, and allowing the parties to understand and relate to the program. Positive feedback was also obtained regarding the group chat system, which allowed students to virtually connect with other members of their cohort in the unprecedented circumstances of social distancing in which anxiety and loneliness levels have increased due to social withdrawal. Student feedback indicated that this group chat function provided some relief in connecting with those facing similar experiences during the pandemic. An innovative passive approach to ensure mental well-being and teach coping mechanisms to the students was taken by Blasco et al. [27] in Brazil. Short videos aimed at addressing problems arising from the pandemic were provided. These videos were adapted from cinema movies, and each one featured a senior teacher speaking about a related subject (e.g., maintaining emotional stability, team-based work, leadership, how to stay focused, etc.). A qualitative analysis of preliminary feedback from the participants showed that the videos addressed problems that were parallel with everyday experience, leaving a positive impact on the participants.

### Combination of support approaches

Both academic and mental support were discussed simultaneously in four studies [26, 28, 29, 35], three of which [28, 29, 35] described the transitioning of previously active mentoring programs to online platforms. Both individual and group activities were used to address the needs of the students. The results of these studies showed students and faculty members to be highly receptive to online methods for mentoring, indicating that they could be as effective as face-to-face mentoring. The programs mainly included career counseling and sessions aimed at reducing social isolation. One study reported that students using mentoring services were less mentally burdened than those who did not partake in mentoring activities [29]. Ashokka et al. [26] provided social support to students who had returned from other countries and/or were quarantined. Support was provided through volunteer groups, peer support systems, and faculty staff, some of whom visited the student dormitories while maintaining personal protection. Relief packages were also dispensed among the students.

## Discussion

A review of the current literature on student support systems during the current COVID-19 pandemic is represented in the present article. Not unexpectedly, all of the included studies employed internet-based infrastructure for supporting students at least in part of their program. Except for some included studies, the majority of reports were not explicitly designed to investigate outcomes of the devised support strategies and merely described the innovations. During the COVID-19 pandemic, more context-appropriate support mechanisms are required to lessen the burden on the students and better prepare for similar future situations [37, 38]. COVID-19-related deaths and morbidities, as well as protective policies implemented by authorities such as social distancing and cessation of in-person activities, have affected mental health negatively and, as a result, led to psychological problems among students [16–19, 39–41]. These repercussions are significant enough to necessitate extensive and direct measures to mitigate the pandemic’s effect on individuals and communities [42].

The COVID-19 pandemic has pushed the experts in the medical education community to develop and disseminate their findings as quickly as possible. This has resulted in a rapid and rather unusual expansion of the literature in this field, reflecting the interest of researchers in the different ways of supporting medical students during this era [36, 43]. Coping with the unexpected changes necessitates educational and emotional support [44–46], which will help to stabilize the current chaos in educational systems. However, to date, the primary focus of universities has mainly been addressing students’ physical health (e.g., cancellation of in-person activities), with mental health often being overlooked.

Medical training is not the same in format across the world. First, there are multiple paths of entering medical school depending on the country. In most countries, students can enter a medical school once they finish secondary school. In North American countries, however, a bachelor’s degree is also required. Second, undergraduate medical education duration varies from place to place. For instance, in the USA, students spend 4 y in undergraduate medical education, while this period lasts for 5 y in Singapore and UK, 6 y in Germany and Brazil, and 7 y in Iran [47, 48]. Mandatory medical service programs before entering residency are also considered in some parts of the world like Iran. Finally, the role of medical students in the COVID-19 era is an area of dispute, with some hospitals forcing them to serve as healthcare workers. Based on variations in undergraduate medical education, appropriate support systems for students should be considered to serve them in their own educational context and culture.

### How can mental health support be addressed?

As described earlier, most of the reviewed studies’ primary concerns were addressing mental health issues. Among the extracted studies, both active and passive measures were used to support students mentally. Whether in groups or individually, online mentoring activities were reported to be as effective as in-person activities in mitigating the mental burden [29]. Also, producing videos and teaching trainees through engaging modalities may be more appreciated by students [27]. While any effort to reduce the psychological burden of students, especially in this period, is noteworthy, it should be remembered that the main approach of universities should be to eliminate the causes of such psychological burden. Uncertainties about being a competent physician, lack of internet access, unfamiliarity with online education service, campus closure, time gaps in different parts of the world in distance education, financial difficulties for students and their families during the pandemic, and a lack of social contact are among issues that have adversely impacted students’ mental health [18, 39, 40, 49–53]. Devising a comprehensive support system that addresses all of the emerging issues can help reduce these burdens. Of note, some students are much more prone to mental health issues than others. Even before the pandemic, students from low-income families, immigrants, and minority groups faced many hardships [9]. As a result of the new problems posed by the pandemic, these people are unquestionably much more fragile than before and need extensive attention [9, 18]. Hence, providing support to vulnerable students in the light of current difficulties is indispensable.

### What is the role of peer support strategies?

Peer support has recently received a lot of attention in medical education [13, 54]. It is a two-way interaction between students from the same discipline that helps them meet their academic objectives. During this process, students gain advice about how to cope with mental and academic difficulties more effectively from senior peers or faculty members [55]. The importance of this type of support in the COVID-19 era is much more significant due to physical distancing restrictions between students. Rastegar Kazerooni et al. achieved positive results through peer mentoring between medical students via social media [33]. Bridson et al. described a number of internet-based peer support services for healthcare professionals worldwide, emphasizing the importance of addressing mental health issues during the pandemic by utilizing this approach [56]. Although this form of assistance is not exclusive to the COVID-19 era, the use of such internet-based support by universities can be highly beneficial given the circumstances of social and physical distancing.

### Infrastructure as a key concern

Although online methods have been suggested in all included studies, it should be borne in mind that using these methods cannot act as a panacea for all problems that arise due to the pandemic, and the tendency for face-to-face support methods prevailed, especially after vaccination. Throughout these volatile times, online teaching has facilitated the continuity of medical training [57–60]. However, both faculty members and students, particularly in less developed parts of the globe, are suffering from a lack of internet and infrastructure access, which has contributed to inequities in the use of online education systems by students [9, 61, 62]. Besides, we should not forget that many clinical students are forced to attend rotations and must live in dormitories. As a result, one of the most critical facets of aiding students at this period is not only paving the way for them to use new educational services, but also providing support in terms of facilities to reduce the current burden. Ashokka et al. reported that providing relief packages to students who were in quarantine or those who came back from abroad can positively impact students [26]. Considering that physical distancing is an effective method to prevent the spread of the disease, medical schools are forced to use online and virtual strategies to support students. This sudden change in the way students are educated will undoubtedly give rise to many problems for universities [37]. Therefore, the service delivery infrastructure in medical schools is bound to change, and further research should be done to determine and assess novel ways of providing support to students.

### What can we do?

Considering the numerous challenges that students face during this period, no one can deny the value of providing them with support. But how can this assistance be provided? Do we adequately support the healthcare workers of the not-so-distant future? Do we help them on the road to becoming those who take action to address a society’s needs? Will part-time and short-term support help to eliminate these shortages? We should aim to answer these questions in future research.

**Fig. 2:**
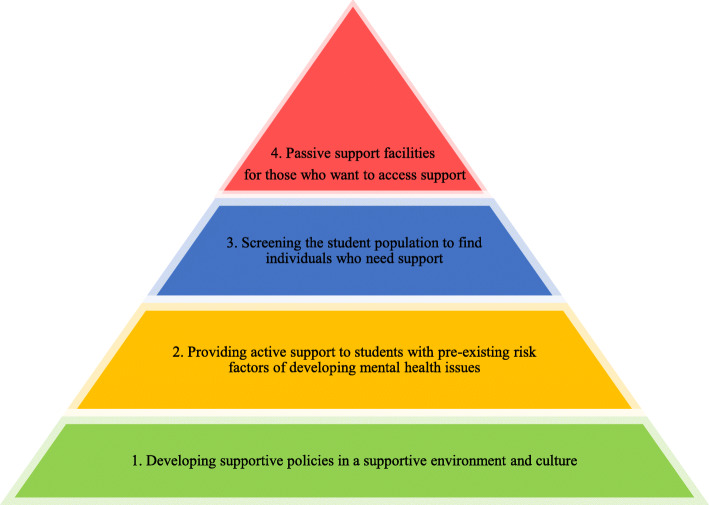
Schema of proposed support system

The mentioned obstacles can be turned into incentives by improving student support programs. We propose a support system consisting of four different levels that actively respond to the concerns of students (Fig. 2). At the first level, supportive policies should be aimed at preventing problems that adversely affect students and foster a supportive culture and environment. At the second level, students with pre-existing conditions that could lead to psychosocial issues should be cared for in order to prevent the development of serious mental conditions. At the third level, authorities should actively find students who are most at risk of suffering from the psychological problems caused by changes related to the pandemic. Also, screening should be employed to identify students who need support. At the fourth level, passive support must also be provided by the universities, and students can use these services if required. Much of what is offered today as student support in medical universities falls into this level. Future research should take into account these various levels of support and help students who are using innovative approaches. While offering support services at each of these levels is valuable, it should be acknowledged that prevention always takes precedence over treatment, and the factors that put pressure on students should be eliminated to the greatest extent possible. The following steps must be taken to implement a system that responds to the students’ needs during this era: (a) a needs assessment should be performed among students; (b) medical education specialists and faculty officials should plan to meet the identified needs; (c) the impacts of the implemented programs should be investigated to ensure their quality.

Of note, universities had valuable systems in place to support students before the pandemic began. With the onset of the pandemic and the implementation of preventive policies, many of these programs were inevitably canceled. However, by taking advantage of digital systems, we can move from conventional to online approaches. As described in some studies, a transition to online strategies for supporting and mentoring students has the potential to be as efficient as previous services [28, 29, 35]. However, the outcomes of the online programs are yet to be evaluated.

This study reviewed new ways to empower and support students during this challenging time. As reported in two previous systematic reviews on medical education developments in response to COVID-19, only 4–6.3% of literature has dealt with student support [36, 43]. In the present research, we considered three studies that were common with the previous reviews [30, 32, 33]. Due to different selection criteria and rapid expansion of the literature, we included seven studies that had not been mentioned in the two previous high-quality reviews.

### Strengths and limitations

As described above, there are several ways of supporting students during these unprecedented times, and it is up to medical faculties around the world to support and empower their students. The reviewed studies present valuable information for medical school authorities who wish to employ different support systems for students during the unforeseeable future. As a limitation, first, there were no randomized controlled trials or high-quality interventions on the efficacy of support systems devised in response to COVID-19. This can be a call for action for researchers to design and implement high-quality interventions based on what we have learned from previous reports. Second, the literature’s infancy resulted in a paucity of relevant articles and, consequently, a small number of included studies. Third, although all reviewed studies hinted at the excellent outcomes of their programs, they mostly described their experience with innovating new support methods rather than comprehensively evaluating the outcomes. Fourth, we narrowed our inclusion criteria to undergraduate medical education, while there may be valuable interventions to support trainees in other disciplines. Finally, we reviewed the interventions described in the literature, while there could be support systems around the world that are yet to be mentioned in publications.

## Conclusion

Medical students are vulnerable during the COVID-19 pandemic but, if adequately supported both mentally and academically, they can help combat the heavy burden imposed by the pandemic on healthcare organizations around the globe. Moreover, we all know that this will not be the last pandemic, so student support and training during the present outbreak can make a strong foundation to comprehend how to fight future instances. Taken together, the results of our review assert that methods of supporting medical students should be adapted to the new circumstances and environments and should provide different levels of support through both online and in-person strategies. As proposed in the studies, online methods for support can be as effective as face-to-face strategies. Codified instructions that facilitate the use of online educational methods for students and faculty members can also help in achieving optimal training. Future research should investigate the question of the extent to which online support can supersede in-person strategies.

## Supplementary Information

**Additional file 1:** Search strategies and Quality appraisal tool.

## Data Availability

Not applicable.

## Abbreviations

COVID-19: Coronavirus Disease 2019
SARS-CoV-2: Severe Acute Respiratory Syndrome Coronavirus 2
