# Supplementary material for: Student support systems for undergraduate medical students during the COVID-19 pandemic: a systematic narrative review of the literature
Source: BMC Med Educ. 2021 Jun 22;21:352. doi: 10.1186/s12909-021-02791-9 (PMC8218568; doi:10.1186/s12909-021-02791-9)
Supplement: Supplementary file 1 — Additional file 1: Search strategies and Quality appraisal tool. [file 12909_2021_2791_MOESM1_ESM.docx]

| Database | Search query |
| --- | --- |
| PubMed | ((pandemics[MeSH Terms]) OR (pandemic*[Title/Abstract]) OR (sars-cov-2[MeSH Terms]) OR (covid-19[MeSH Terms]) or (coronavirus[MeSH Terms]) OR (coronavirus[Title/Abstract]) OR (covid*[Title/Abstract]) OR (“SARS-Cov-2*”[Title/Abstract]) OR (“2019-nCoV*”[Title/Abstract]) OR (“2019 novel coronavirus”[Title/Abstract]) OR (“coronavirus disease*”[Title/Abstract])) AND ((medical education[MeSH Terms]) or (“medical education”[Title/Abstract]) or (medical student*[MeSH Terms]) or (“medical student*”[Title/Abstract]) or (undergraduate medical education[MeSH Terms]) or (“undergraduate medical education”[Title/Abstract]) or (medical school*[MeSH Terms]) or (“medical school*”[Title/Abstract]) or (clinical clerkship*[MeSH Terms]) or (“clinical clerkship*”[Title/Abstract]) or (“clinical rotation*”[Title/Abstract]) OR (“clinical educat*”[Title/Abstract]) OR (“clinical training”[Title/Abstract])) AND ((mentor[MeSH Terms]) or (mentoring[MeSH Terms]) OR (mentor*[Title/Abstract]) OR (mentee*[Title/Abstract]) OR (coach*[Title/Abstract]) OR (counsel*[Title/Abstract]) OR (“student support”[Title/Abstract]) OR (“support system”[Title/Abstract]) OR (peer[Title/Abstract]) OR (advis*[Title/Abstract]) OR (guide[Title/Abstract]) OR (confidant[Title/Abstract]) OR (consult*[Title/Abstract]) OR (cheerleader[Title/Abstract]) OR (trainer[Title/Abstract]) OR (tutor[Title/Abstract]) OR (helper[Title/Abstract]) OR (aid[Title/Abstract]) OR (director[Title/Abstract]) OR (instruct*[Title/Abstract]) OR (guru[Title/Abstract]) or (“pastoral support”[Title/Abstract])) |
| Embase | ('pandemic'/exp OR ‘pandemic*’:ab,ti OR 'coronavirus disease 2019'/exp OR 'Severe acute respiratory syndrome coronavirus 2'/exp OR 'coronavirus'/exp OR coronavirus:ab,ti OR covid*:ab,ti OR 'sars cov 2*':ab,ti OR '2019 ncov*':ab,ti OR '2019 novel coronavirus':ab,ti OR 'coronavirus disease*':ab,ti) AND ('medical education'/exp OR 'medical education':ab,ti OR 'medical student'/exp OR 'medical student':ab,ti OR 'undergraduate medical education':ab,ti OR 'medical school'/exp OR 'medical school':ab,ti OR 'clinical education'/exp OR 'clinical educat*':ab,ti OR ‘clinical rotation’:ab,ti OR ‘clinical clerkship’:ab,ti OR ‘clinical training’:ab,ti) AND ('mentor'/exp OR 'mentor*':ab,ti OR 'mentoring'/exp OR 'counseling'/exp OR 'peer group'/exp OR 'peer':ab,ti OR mentee*:ab,ti OR coach*:ab,ti OR counsel*:ab,ti OR 'student* support':ab,ti OR 'support system':ab,ti OR advis*:ab,ti OR guide:ab,ti OR confidant:ab,ti OR consult*:ab,ti OR cheerleader:ab,ti OR trainer:ab,ti OR tutor:ab,ti OR helper:ab,ti OR aid:ab,ti OR director:ab,ti OR instructor:ab,ti OR guru:ab,ti OR ‘pastoral support’:ab,ti) |
| Scopus | (INDEXTERMS( "coronavirus" OR "covid-19" OR "sars-cov-2") OR TITLE-ABS("coronavirus" or "covid*" OR "SARS-Cov-2*" OR "2019-nCoV*" or "2019 novel coronavirus*" or "coronavirus disease*")) AND (INDEXTERMS("medical education" OR "medical student*" OR "undergraduate medical education" OR "medical school*" OR "clinical clerkship*") OR TITLE-ABS("medical education" OR "medical student*" OR "undergraduate medical education" OR "medical school*" OR " clerkship*" OR "clinical educat*" OR "clinical rotation" OR "clinical training")) AND (INDEXTERMS( "mentor*" ) OR TITLE-ABS("mentor*" or "mentee*" or "coach*" or "counsel*" or "student* support" or "support system" or "peer" or "advis*" or "guide" or "confidant" or "consult*" or "cheerleader" or "trainer" or "tutor" or "helper" or "aid" or "director" or "instructor" or "guru" or "pastoral support")) |
| WOS | (TS=("coronavirus" or "covid*" OR "SARS-Cov-2*" OR "2019-nCoV*" or "2019 novel coronavirus" or "coronavirus disease*")) AND (TS=("medical education" or "medical student*" or "undergraduate medical education" or "medical school*" or "clerkship*" or "clinical educat*" or “clinical rotation” or “clinical training”)) AND (TS=("mentor*" or "mentee*" or "coach*" or "counsel*" or "student* support" or "support system" or "peer" or "advis*" or "guide" or "confidant" or "consult*" or "cheerleader" or "trainer" or "tutor" or "helper" or "aid" or "director" or "instructor" or "guru" or "pastoral support")) |
| ERIC | (coronavirus or covid* OR SARS-Cov-2 OR 2019-nCoV) AND (medical education or medical student or undergraduate “medical education” or medical school or internship or “clinical educat*” OR “clinical training” OR “clinical rotation” or clerkship*) AND (mentor* or mentee* or coach* or counsel* or student* support or support system or peer) |
| Cochrane | ("coronavirus" or "covid*" OR "SARSCov2*" OR "2019nCoV*" or "2019 novel coronavirus" or "coronavirus disease*"):ti,ab,kw AND ("medical education" or "medical student" or "undergraduate medical education" or "graduate medical education" or "medical school" or "clerkship*" or "clinical educat*" or “clinical training” or “clinical rotation”):ti,ab,kw AND ("mentor*" or "mentee*" or "coach*" or "counsel*" or "student* support" or "support system" or "peer" or "advis*" or "guide" or "confidant" or "consult*" or "cheerleader" or "trainer" or "tutor" or "helper" or "aid" or "director" or "instructor" or "guru" or "pastoral support"):ti,ab,kw |

Quality appraisal tool

| Indicator | Description |
| --- | --- |
| 1-Research question | Is the research question(s) or hypothesis clearly stated? |
| 2-Study subjects | Is the subject group appropriate for the study being carried out (number, characteristics, selection, and homogeneity)? |
| 3-Data collection methods | Are the methods used (qualitative or quantitative) reliable and valid for the research question and context? |
| 4-Completeness of data | Have subjects dropped out? Is the attrition rate less than 50%? For questionnaire based studies, is the response rate acceptable (60% or above)? |
| 5-Control for confounding | Have multiple factors/variables been removed or accounted for where possible? |
| 6-Analysis of results | Are the statistical or other methods of results analysis used appropriate? |
| 7-Conclusions | Is it clear that the data justify the conclusions drawn? |
| 8-Reproducibility | Could the study be repeated by other researchers? |
| 9-Prospective | Does the study look forwards in time (prospective) rather than backwards (retrospective)? |
| 10-Ethical issues | Were all relevant ethical issues addressed? |
| 11-Triangulation | Were results supported by data from more than one source? |

Buckley S, Coleman J, Davison I, Khan KS, Zamora J, Malick S, Morley D, Pollard D, Ashcroft T, Popovic C, Sayers J. The educational effects of portfolios on undergraduate student learning: a Best Evidence Medical Education (BEME) systematic review. BEME Guide No. 11. Med Teach. 2009 Apr;31(4):282-98. doi: 10.1080/01421590902889897. PMID: 19404891.
